# Household wealth is associated with child stunting but not wasting: Evidence from the 2024 Nigeria Demographic and Health Survey

**DOI:** 10.1371/journal.pone.0357124

**Published:** 2026-08-27

**Authors:** John Adeleke

**Affiliations:** Department of Sociology, University of California, Merced, California, United States of America; Innovative Aid, CANADA

## Abstract

Child undernutrition remains a significant population health challenge in Nigeria and sub-Saharan Africa. Existing research has established an association between socioeconomic factors and child undernutrition, but less attention has been paid to whether the socioeconomic gradient is similar across indicators that capture different underlying processes. Drawing on fundamental cause theory, this study examines the association between household wealth and child stunting and wasting in Nigeria, and the extent to which that association is attenuated by other socioeconomic and geographic factors. Using the 2024 Nigeria Demographic and Health Survey (n = 9,410 children for stunting; n = 9,464 for wasting), Survey-weighted logistic regression models were fitted, using stepwise model building and joint tests of interaction. Household wealth was associated with stunting in a graded pattern but showed no association with wasting. The findings suggest that chronic and acute undernutrition may not share the same social determinants and may call for different interventions.

## Background

### Child stunting and wasting in Nigeria

Stunting and wasting are major indicators of child undernutrition and mortality [1–3]. Stunting, low height-for-age, reflects chronic or recurrent undernutrition over a long period and is linked to impaired physical growth and cognitive development that can be irreversible [4]. Wasting, low weight-for-height, reflects acute weight loss from recent illness or insufficient food intake [5, 6]. The two indicators capture different processes, one slow and cumulative and the other acute and often episodic. This distinction is central to this study. In Nigeria, socioeconomic factors are associated with stunting, wasting, and underweight, as well as with child mortality and infectious illness (Nwanze et al., 2023). Using decomposition analysis, Nwosu and Ataguba [7] report a stunting concentration index of about 0.33, indicating that stunting is concentrated among children in low-wealth households.

Analyses of trends over time find that socioeconomic inequalities in child undernutrition have persisted and, in some cases, widened [8]. Such wealth-related inequalities are well documented across sub-Saharan Africa, though their magnitude varies by context [2]. Child undernutrition is often framed in terms of immediate, underlying, and basic causes. Immediate causes include inadequate diet and infection; underlying causes include food insecurity, poor maternal health, inadequate care, and limited health services; and basic causes include poverty, education, geography, and social status (Le et al., 2020). Fundamental cause theory directs attention to these basic factors. The association between these basic causes and indicators of child undernutrition, such as stunting and wasting, remains unclear, and this study asks whether the relationship between socioeconomic status and child undernutrition depends on the specific indicator.

### Socioeconomic and geographic context

Nigeria’s wealth distribution is highly unequal, and poorer households often lack access to clean water, electricity, sanitation, and adequate shelter [9,10]. Because income and consumption are difficult to measure reliably where informal and seasonal work is common, researchers and national surveys rely on a household wealth index based on assets and living conditions. Household wealth is less sensitive to short-term shocks than income and better captures long-term living conditions, which makes it a stable indicator of socioeconomic position for studying child health [11,12]. Mothers’ education is also strongly linked to child health. Educated mothers tend to be more informed about hygiene, nutrition, and navigating the health system, and are more likely to practice exclusive breastfeeding and appropriate complementary feeding [13,14].

Education does not always translate into economic power or systemic access, particularly where patriarchal norms and early marriage constrain women’s autonomy [15]. Its influence on child health may therefore depend on wealth and context. These socioeconomic patterns are geographically structured. Colonial and post-colonial development in Nigeria favored the south, while the north received less investment in education, infrastructure, and health services [16,17]. Northern regions show lower female educational attainment, higher poverty, and greater exposure to conflict, especially in the Northeast [10,18]. Rural-urban gaps compound regional ones: rural women receive less antenatal care than urban women [19,20], and UNICEF reports that children in northern Nigeria are worst affected by stunting, wasting, and mortality [21]. Wealth, education, and geography are thus overlapping dimensions of the same structure of disadvantage.

### Fundamental cause theory

Fundamental cause theory holds that socioeconomic status shapes health because it provides access to flexible resources, including money, knowledge, and social connections, that can be used to avoid health risks and limit their consequences [22]. A fundamental cause affects multiple health outcomes through multiple mechanisms and retains its effect as specific mechanisms change, because resources can be redeployed to pathways that matter at a given time. The theory has been applied mainly to persistent disparities in high-income countries, and its application in low- and middle-income settings remains limited. A few studies apply the theory to the sub-Saharan Africa context. Langa [23] finds that wealthier and more educated women in Tanzania have greater access to skilled antenatal and delivery care. Focusing on children under five in Madagascar, [24] show that parental education and wealth strongly predict vaccination uptake and malaria-prevention knowledge and use. These studies suggest that socioeconomic resources shape access to proximate, health-protective factors even in low-income settings.

Applying this lens to household wealth and child undernutrition in Nigeria would help move beyond documenting disparities toward asking why they endure. Household wealth has the characteristics of a fundamental cause: it is associated with many child health outcomes, operates through several pathways, including nutrition, water and sanitation, and household energy, and provides flexible resources that help households respond to health threats and achieve better health outcomes for their children. Mothers’ education is a related but distinct resource that may interact with wealth rather than substitute for it [22]. Because the data are cross-sectional, this study cannot test two of the theory’s defining claims: that the effect of wealth persists over time, and that it continues even as the specific mechanisms linking wealth to health change. Fundamental cause theory is therefore used as an interpretive lens rather than a hypothesis to be confirmed. The theory yields one testable expectation. If flexible resources matter most for health outcomes that build up gradually through many pathways, then the wealth gradient should be steeper for stunting, a chronic condition, than for wasting, an acute condition that results from sudden shocks such as illness or short-term food shortage.

### Study aims and hypotheses

Building on this expectation, this study examines the association between household wealth and child stunting and wasting in Nigeria, and whether that association differs between the two indicators after accounting for mothers’ education, region, urban-rural and child characteristics. Two hypotheses are tested:

H1: Children from wealthier households will have lower odds of experiencing stunting and wasting than children in the poorest households.

H2: The association between household wealth and each outcome will attenuate after adjustment for mothers’ education, region, urban-rural, and child characteristics.

## Methods

### Study design and data source

This study utilized secondary data from the 2024 Nigeria Demographic and Health Survey (NDHS), a nationally representative household survey [25]. The NDHS follows a stratified, two-stage cluster sampling design. Enumeration areas were first selected with probability proportional to size within sampling strata formed by the cross-classification of the 36 states and the Federal Capital Territory by urban and rural residence; households were then selected systematically within each enumeration area. Survey data on household characteristics and on the health and nutrition of women aged 15–49 and their children under five were collected, including anthropometric measurements. This analysis used the Children’s Recode (KR) file, which contains one record per living child born in the five years before the survey.

### Study population and sample

The study population comprised children under five years of age. Because the outcomes are anthropometric, the analysis was restricted to children with valid measurements of the indicators of interest. The DHS Program computed stunting and wasting using height-for-age and weight-for-height z-scores, respectively. For each indicator, children whose z-score was missing (mainly because height or weight was not measured, for example for children who were absent or ill at the time of the visit) were excluded, and those with biologically implausible values outside ±6 SD, following standard DHS practice. Applying these criteria yielded analytic samples of 9,410 children for stunting and 9,464 for wasting. Missing data among the covariates were negligible and did not materially affect the sample size, so a complete-case analysis was used for each outcome.

### Dependent variables: stunting and wasting

The two dependent variables of interest are stunting and wasting, derived from children’s anthropometric measurements using World Health Organization child growth standards. Stunting was measured using the height-for-age z-score (HAZ), and children were classified as stunted if their HAZ was below −2 standard deviations from the reference population median. Wasting was based on weight-for-height z-score (WHZ), with children classified as wasted if their WHZ was below −2 standard deviations. These are binary variables with zero representing not stunted or wasted, and one representing stunted or wasted.

### Independent variable: household wealth

Household wealth index is a principal component analysis-derived composite of over 100 indicators of asset ownership (e.g., electronics, housing materials, water, and sanitation access), standardized to a mean of zero and a standard deviation of one. The wealth index was retained in its standard five-quintile form, from poorest to richest, with the poorest quintile as the reference category.

### Covariates

Covariates were selected based on established determinants of child undernutrition and this study’s theoretical framework [8,22,26]. Mothers’ education was categorized as no education, primary, secondary, or higher (reference: no education). Place of residence was rural or urban (reference: rural). Geopolitical region comprised the six Nigerian regions: North Central, North East, North West, South East, South South, and South West (reference: North Central). The fully adjusted model additionally included child age in months to capture the non-linear relationship between age and anthropometric status, and child sex (reference: male). Child age and sex are associated with stunting and wasting and are measured in the recode file.

### Statistical analysis

Since each outcome is binary (stunted versus not stunted; wasted versus not wasted), binary logistic regression was used, and odds ratios with 95% confidence intervals were reported. All analyses were survey-weighted and accounted for the complex sampling design, with the primary sampling unit, sampling stratum, and child sampling weight specified so that standard errors reflect clustering and stratification and the estimates are representative of Nigerian children under five [25]. Sample characteristics and the weighted prevalence of each outcome across categories were described. For each outcome, models were fitted in sequence to show how the wealth association changed as additional factors were taken into account. Model 1 included wealth only; Model 2 added mothers’ education; Model 3 added residence; Model 4 added region; and Model 5, the fully adjusted model, added child age and sex. To present the gradient on an interpretable scale, the predicted probability of each outcome by wealth quintile was estimated from the unadjusted and fully adjusted models (Williams, 2012). Whether the wealth gradient varied by mothers’ education, residence, and region was then tested by adding interaction terms between wealth and each covariate to the fully adjusted model. Because these interactions involve many parameters across two outcomes, they were treated as exploratory and assessed each with a single joint Wald test of all its product terms, rather than interpreting individual cross-product coefficients. All analyses were performed in Stata 19.5.

### Ethical considerations

This study analyzed de-identified, publicly available secondary data and involved no contact with participants. The original NDHS protocol, including informed-consent procedures, was reviewed and approved by the National Health Research Ethics Committee of Nigeria and the Institutional Review Board of ICF. Permission to use the data was granted by the DHS Program. Because the analysis used only de-identified secondary data, it did not constitute human-subjects research requiring additional institutional review.

## Results

### Sample characteristics and prevalence of stunting and wasting

Table 1 presents the weighted characteristics of the analytic samples and the prevalence of each outcome. Overall, 39.1% of children under five were experiencing stunting) and 8.3% were experiencing wasting. The prevalence of stunting declined steadily across wealth quintiles, from 55.6% among children in the poorest households to 14.8% among those in the richest, and fell similarly across levels of mothers’ education, from 55.1% among children of mothers with no education to 14.0% among those whose mothers had higher education. Stunting was more common in rural than urban areas (47.4% versus 27.8%) and was concentrated in the northern regions, ranging from 52.4% in North Central and 51.8% in North East to 17.9% in South South. Wasting showed a different pattern. Its prevalence varied little across wealth quintiles, from 9.5% in the poorest to 8.7% in the richest, with no consistent trend, and the difference across quintiles was not statistically significant (p = 0.086). Wasting also did not differ significantly by mothers’ education (p = 0.101). It varied modestly by residence (7.7% rural versus 9.4% urban, p = 0.030) and by region (from 6.5% in North West to 11.8% in South South, p = 0.002), though without the north–south differences seen for stunting.

**Table 1:** Weighted sample characteristics and prevalence of child stunting and wasting, Nigeria DHS 2024.

Characteristic	Weighted %	Stunted, %	p-value	Wasted, %	p-value
**Household wealth**			<0.001		0.086
Poorest	24.4	55.6		9.5	
Poorer	22.7	51.7		7.6	
Middle	20.2	40.6		6.9	
Richer	17.6	30.2		9.4	
Richest	15.1	14.8		8.7	
**Mother’s education**			<0.001		0.101
No education	47.0	55.1		8.8	
Primary	11.9	40.5		7.7	
Secondary	31.2	28.8		9.0	
Higher	9.9	14.0		6.3	
**Residence**			<0.001		0.030
Rural	61.6	47.4		7.7	
Urban	38.4	27.8		9.4	
**Geopolitical region**			<0.001		0.002
North Central	39.5	52.4		8.4	
North East	19.9	51.8		8.3	
North West	16.4	36.6		6.5	
South East	7.2	19.6		7.2	
South South	7.4	17.9		11.8	
South West	9.7	21.1		10.4	
**Overall**	**100.0**	**39.1**		**8.3**	

### Household wealth and stunting

Table 2 shows odds ratios from the stunting models. In the unadjusted model, the odds of stunting decreased at each step up the wealth distribution relative to the poorest quintile. Children from poorer households had 15% lower odds of stunting; those from the middle quintile had 46% lower odds; those from the richer quintile had 65% lower odds; and those from the richest quintile had 86% lower odds of stunting. Adjustment for mothers’ education attenuated these estimates, reducing the difference between the richest and poorest quintiles from 86% to 70% lower odds. Further adjustment for residence and region attenuated it to 58% lower odds. In the fully adjusted model, which included child age and sex, the wealth association remained: children from the middle quintile had 19% lower odds of stunting, those from the fourth quintile 31% lower odds, and those from the richest quintile 61% lower odds than children from the poorest households. The second quintile did not differ significantly from the poorest. In the fully adjusted model, mothers’ education remained inversely associated with stunting at all levels. Urban residence was associated with 22% lower odds of stunting. Relative to North Central, the odds of stunting were 40% lower in North West, 68% lower in South East, 64% lower in South South, and 52% lower in South West; North East did not differ significantly.

**Table 2:** Survey-weighted logistic regression of child stunting on household wealth, Nigeria DHS 2024 (odds ratios).

Variable	Model 1	Model 2	Model 3	Model 4	Model 5
**Household wealth (ref: Poorest)**					
Poorest	1.00 (ref)	1.00 (ref)	1.00 (ref)	1.00 (ref)	1.00 (ref)
Poorer	0.85 (0.74–0.99)*	0.97 (0.83–1.13)	0.98 (0.84–1.14)	1.02 (0.87–1.19)	1.00 (0.84–1.19)
Middle	0.54 (0.46–0.65)***	0.74 (0.62–0.88)***	0.77 (0.64–0.92)**	0.84 (0.70–1.01)	0.81 (0.67–0.98)*
Richer	0.35 (0.29–0.41)***	0.57 (0.47–0.70)***	0.63 (0.51–0.77)***	0.72 (0.59–0.89)**	0.69 (0.55–0.86)***
Richest	0.14 (0.11–0.18)***	0.30 (0.23–0.39)***	0.33 (0.25–0.45)***	0.42 (0.31–0.57)***	0.39 (0.28–0.54)***
**Mother’s education (ref: No education)**					
Primary		0.65 (0.53–0.80)***	0.65 (0.53–0.80)***	0.80 (0.66–0.98)*	0.80 (0.65–0.98)*
Secondary		0.51 (0.43–0.60)***	0.52 (0.44–0.61)***	0.75 (0.63–0.89)**	0.74 (0.62–0.88)***
Higher		0.30 (0.22–0.41)***	0.31 (0.23–0.42)***	0.42 (0.31–0.57)***	0.40 (0.29–0.55)***
**Residence (ref: Rural)**					
Urban			0.84 (0.71–1.00)*	0.81 (0.68–0.96)*	0.78 (0.65–0.93)**
**Geopolitical region (ref: North Central)**					
North East				0.95 (0.78–1.15)	0.96 (0.79–1.18)
North West				0.63 (0.52–0.76)***	0.60 (0.49–0.74)***
South East				0.34 (0.27–0.42)***	0.32 (0.25–0.40)***
South South				0.37 (0.29–0.48)***	0.36 (0.28–0.47)***
South West				0.49 (0.38–0.64)***	0.48 (0.36–0.63)***
**Child characteristics**					
Age, months					1.11 (1.09–1.12)***
Age², months					1.00 (1.00–1.00)***
Female (ref: Male)					0.75 (0.67–0.83)***
**Observations (n)**	**9410**	**9410**	**9410**	**9410**	**9410**

Odds ratio (95% confidence intervals);* p < 0.05, ** p < 0.01, *** p < 0.001.

### Household wealth and wasting

Table 3 presents odds ratios from the wasting models. In the unadjusted model, the joint test of household wealth was not significant. No quintile differed significantly from the poorest except the middle quintile, which had 29% lower odds of wasting. This pattern was consistent across all models. In the fully adjusted model, children from the poorer quintile had 20% lower odds of wasting; those from the middle quintile 30% lower odds; those from the richer quintile 5% lower odds; and those from the richest quintile 9% lower odds; and only the middle quintile estimate reached significance. Among covariates in the fully adjusted model, higher maternal education was associated with 51% lower odds of wasting, while primary and secondary education were not statistically significant. Urban residence was associated with 30% higher odds of wasting, and South South with 61% higher odds relative to North Central.

**Table 3:** Survey-weighted logistic regression of child wasting on household wealth, Nigeria DHS 2024 (odds ratios).

Variable	Model 1	Model 2	Model 3	Model 4	Model 5
**Household wealth (ref: Poorest)**					
Poorest	1.00 (ref)	1.00 (ref)	1.00 (ref)	1.00 (ref)	1.00 (ref)
Poorer	0.78 (0.58–1.04)	0.79 (0.59–1.08)	0.79 (0.58–1.07)	0.79 (0.58–1.07)	0.80 (0.58–1.08)
Middle	0.71 (0.53–0.94)*	0.75 (0.55–1.02)	0.70 (0.51–0.95)*	0.69 (0.50–0.96)*	0.70 (0.51–0.97)*
Richer	0.99 (0.75–1.29)	1.10 (0.81–1.50)	0.95 (0.68–1.32)	0.92 (0.66–1.30)	0.95 (0.67–1.34)
Richest	0.90 (0.69–1.19)	1.18 (0.84–1.66)	0.98 (0.68–1.42)	0.89 (0.60–1.32)	0.91 (0.61–1.36)
**Mother’s education (ref: No education)**					
Primary		0.87 (0.64–1.18)	0.85 (0.63–1.16)	0.81 (0.59–1.13)	0.80 (0.58–1.11)
Secondary		0.93 (0.72–1.20)	0.91 (0.70–1.17)	0.83 (0.61–1.11)	0.79 (0.58–1.07)
Higher		0.56 (0.38–0.81)**	0.54 (0.37–0.79)**	0.50 (0.34–0.75)***	0.49 (0.33–0.74)***
**Residence (ref: Rural)**					
Urban			1.33 (1.07–1.65)**	1.25 (1.00–1.57)	1.30 (1.03–1.63)*
**Geopolitical region (ref: North Central)**					
North East				0.94 (0.71–1.25)	0.90 (0.68–1.20)
North West				0.82 (0.60–1.11)	0.80 (0.59–1.09)
South East				0.98 (0.69–1.39)	0.99 (0.70–1.40)
South South				1.63 (1.12–2.38)**	1.61 (1.10–2.35)*
South West				1.33 (0.88–2.00)	1.28 (0.84–1.95)
**Child characteristics**					
Age, months					0.98 (0.96–1.00)
Age², months					1.00 (1.00–1.00)
Female (ref: Male)					0.90 (0.76–1.06)
**Observations (n)**	**9464**	**9464**	**9464**	**9464**	**9464**

Odds ratio (95% confidence intervals);* p < 0.05, ** p < 0.01, *** p < 0.001.

### Predicted probabilities of stunting and wasting by household wealth

**Fig 1:**
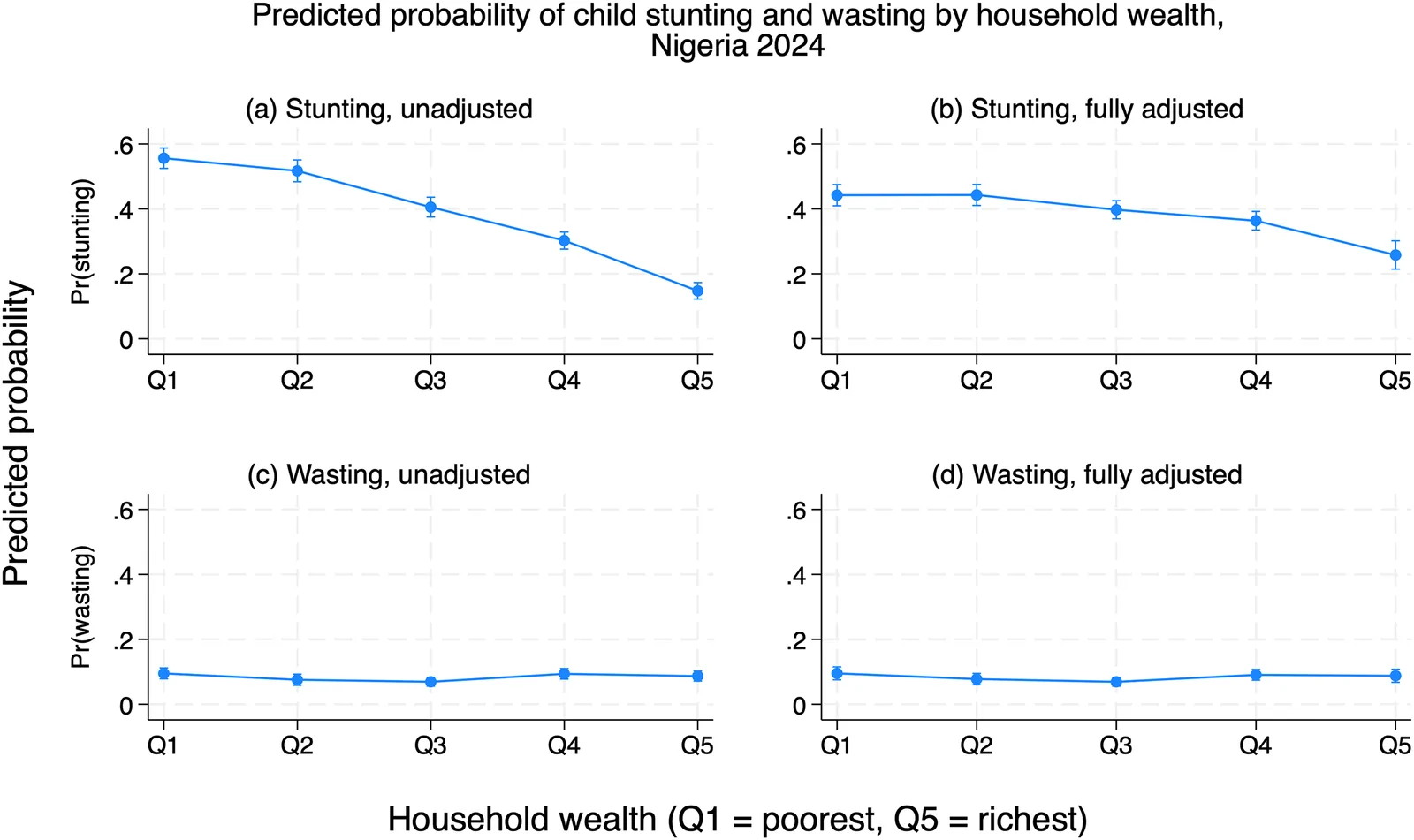
Predicted probability of child stunting and wasting by household wealth quintile, Nigeria DHS 2024.

Fig 1 below shows the predicted probabilities of stunting and wasting across wealth quintiles, from the unadjusted and fully adjusted models. For stunting, the predicted probability decreased steadily across the wealth distribution. In the unadjusted model, it fell from 55.6% in the poorest quintile to 14.8% in the richest quintile. After full adjustment, the gradient was flatter but still present, falling from 44.2% to 25.8%. For wasting, the predicted probabilities were flat across the wealth distribution in both models. They ranged from 6.9% to 9.5% in the unadjusted model and from 6.9% to 9.5% after full adjustment, with no graded pattern and overlapping confidence intervals across all five quintiles

### Interaction between household wealth and covariates

Table 4 shows whether the wealth gradient is the same for all children or depends on a mother’s education, region, or urban-rural residence. This matters because national-level estimates can mask variation, and a single average may understate the gradient in some settings while overstating it in others. For stunting, the magnitude of the wealth gradient varied by place of residence and geopolitical region. In other words, the extent to which a household’s wealth is associated with a child’s odds of stunting varies across Nigeria; it depends on whether the family lives in a rural or urban area and in which region. The gradient did not differ across levels of mothers’ education, meaning that wealth was associated with stunting to a similar degree regardless of a child’s mother’s education, even though education was itself associated with stunting in the main models. For wasting, the wealth gradient did not differ by mothers’ education, residence, or region. This follows from the main results because wealth showed no association with wasting overall and there was no gradient for these interactions to modify.

**Table 4:** Joint tests of interaction between household wealth on stunting and wasting, Nigeria DHS 2024.

Interaction	Stunting F (df)	p-value	Wasting F (df)	p-value
Household wealth × mother’s education	1.61 (12)	0.083	1.03 (11)	0.421
Household wealth × residence	3.63 (4)	0.006	1.35 (4)	0.248
Household wealth × geopolitical region	2.73 (20)	<0.001	1.39 (20)	0.118

Numerator degree of freedom reported and denominator degree of freedom ranged from 1,250–1,266; p-value.

## Discussion

This study examined whether household wealth is associated with child stunting and wasting in Nigeria and whether the socioeconomic difference is similar for both indicators. It is not. Wealth was associated with stunting, a measure of chronic undernutrition, in a graded manner that persisted after adjustment for educational and geographic factors, whereas wasting, a measure of acute undernutrition, showed no wealth gradient at any stage. This divergence is one of this study’s findings. The socioeconomic gradient in stunting is consistent with existing literature. Nwosu and Ataguba [7] find stunting heavily concentrated among children in poorer households, and Akombi et al. [8] show that socioeconomic inequalities in child undernutrition persisted, and in some cases widened, across successive survey rounds. These results extend this body of knowledge in two ways. Retaining the full wealth quintiles shows that the association is graded rather than a contrast between the poorest and everyone else, which is what one expects when resources are converted into health advantage continuously rather than at a single threshold. Adjusting stepwise then separates the part of that gradient shared with other dimensions of socioeconomic position from the part that is not.

Mothers’ education accounted for a large share of it, which fits what is known about how education functions in this context. Educated mothers are better positioned to secure adequate and timely feeding, recognize illness, and utilize health services [13,14], and education and wealth are closely intertwined in Nigeria, where household resources shape whether girls remain in school at all [15]. The attenuation was nonetheless incomplete. A wealth gradient remained after accounting for education and geography, and education retained an independent association, so the two are better understood as overlapping but non-substitutable resources than as a single pathway. The absence of a wealth gradient in wasting indicates that acute undernutrition in Nigeria is distributed across socioeconomic positions rather than concentrated at its lower end. This becomes intelligible once the two indicators are understood as products of different processes rather than as milder and severer versions of one another [6]. Stunting accumulates over months and years through repeated infections, sustained shortfalls in diet quality, and inadequate water, sanitation, and care [4], each of which is sensitive to household resources and rewards the continuous deployment of these resources. Wasting reflects recent weight loss following illness, seasonal food gaps, or disruption to feeding [5]. This suggests that a short, largely episodic process leaves accumulated resources little room to operate, and a wealthier household is not obviously protected against a diarrheal episode or a lean-season shortfall in the weeks preceding measurement. Education remained associated with wasting, whereas wealth was not, perhaps because responding to acute illness depends more on recognizing it early than on paying for care. This rests on a single association and requires direct testing in future studies.

The variation in wealth associated with stunting differed by place of residence and region, though not by mothers’ education level. How much a household’s wealth is associated with a child’s odds of stunting therefore depends on where that household is located, which is expected in a country whose regions differ sharply in the infrastructure, health services, and educational opportunity through which resources can be converted into health advantage [10,17]. The joint interaction tests establish that this variation exists without identifying its direction, and the interactions were specified as exploratory. Two patterns nonetheless complicate the conventional north-south disparities. The regional differences in stunting did not simply reproduce the geographic gradient once wealth, education, and residence were taken into account, suggesting that much of the observed north-south difference reflects the uneven distribution of those resources rather than region as such [27]. Wasting, meanwhile, ran counter to stunting in part, appearing somewhat more often in urban areas and in one southern region despite comparatively favorable socioeconomic indicators. Taken together with the null wealth gradient, this reinforces the point that acute undernutrition is governed by determinants not well captured by socioeconomic position and not measured here, including local food systems, disease environments, and seasonality. If these associations reflect real differences in how the two conditions arise, they suggest that chronic and acute undernutrition may call for different interventions. A wealth gradient in stunting that persists after adjustment implies that educational expansion and geographic targeting are unlikely to close it on their own, and that interventions to address the economic condition of poor households remain relevant. The absence of a gradient in wasting implies, more tentatively, that screening and treatment targeted by household poverty alone would miss a substantial share of affected children. Both follow from an observational pattern rather than a demonstrated effect.

The study’s strengths lie in its data and design: large, nationally representative samples collected under standardized DHS protocols, retention of the full wealth quintiles, and the examination of two distinct indicators, which permit a contrast between the chronic and acute undernutrition. This study is not without its limitations. The NDHS is cross-sectional, so the associations reported here are not causal effects and do not meet the criteria set out by the fundamental cause theory. Although the models adjust for mothers’ education, residence, region, and the child’s age and sex, other established determinants are not available in the Children’s Recode without substantial loss of cases, among them dietary diversity, household food insecurity, water and sanitation, birth spacing, low birth weight, and caregiver mental health, so residual confounding remains possible. This matters most for wasting, where an unmeasured determinant such as recent illness or seasonal food availability could be consequential where socioeconomic position is not. The analysis accounts for stratification, clustering at the primary sampling unit, and sampling weights, but does not model the nesting of children within households, which future work should address. The interaction tests are exploratory and provisional. Finally, anthropometric measurements were unavailable for a substantial proportion of children in the survey file, and the analytic samples comprise only those measured; if measurement was related to nutritional status, the prevalence estimates could be affected.

## Conclusion

Household wealth was associated with chronic undernutrition in Nigeria but not with acute undernutrition. The gradient in stunting was steep and substantially attenuated once mothers’ education and geographic factors were accounted for, yet it persisted. It also varied by residence and region, so the association does not hold uniformly across the country. Wasting showed no wealth gradient at any stage. These findings are consistent with the premise that flexible socioeconomic resources matter most for outcomes that accumulate gradually through many pathways, and they identify a boundary to it. Because the analysis is cross-sectional, these are associations rather than demonstrated effects, and the fundamental-cause interpretation is offered as a lens rather than a tested claim. The broader implication is that stunting and wasting, though routinely reported side by side as indicators of the same problem, may not share the same social determinants.

## References

[1] Adewuyi EO, Zhao Y, Lamichhane R. Risk factors for infant mortality in rural and urban Nigeria: evidence from the national household survey. Scand J Public Health. 2017;45(5):543–54. doi: 10.1177/1403494817696599 28355963

[2] Baye K, Laillou A, Chitweke S. Socio-Economic Inequalities in Child Stunting Reduction in Sub-Saharan Africa. Nutrients. 2020;12(1):253. doi: 10.3390/nu12010253 31963768 PMC7019538

[3] Tamir TT, Gezhegn SA, Dagnew DT, Mekonenne AT, Aweke GT, Lakew AM. Prevalence of childhood stunting and determinants in low and lower-middle income African countries: Evidence from standard demographic and health survey. PLoS One. 2024;19(4):e0302212. doi: 10.1371/journal.pone.0302212 38662745 PMC11045052

[4] Obasohan PE, Walters SJ, Jacques RM, Khatab K. The Risk Factors Associated with the Prevalence of Multimorbidity of Anaemia, Malaria, and Malnutrition among Children Aged 6-59 Months in Nigeria. Int J Environ Res Public Health. 2024;21(6):765. doi: 10.3390/ijerph21060765 38929011 PMC11203752

[5] Bekelman TA. Stunting and Wasting. In: Trevathan W, Cartmill M, Dufour D, Larsen C, O Rourke D, Rosenberg K, et al., editors. The International Encyclopedia of Biological Anthropology. Wiley. 2018:1–2.

[6] Thurstans S, Sessions N, Dolan C, Sadler K, Cichon B, Isanaka S, et al. The relationship between wasting and stunting in young children: A systematic review. Matern Child Nutr. 2022;18(1):e13246. doi: 10.1111/mcn.13246 34486229 PMC8710094

[7] Nwosu CO, Ataguba JE-O. Explaining changes in wealth inequalities in child health: The case of stunting and wasting in Nigeria. PLoS One. 2020;15(9):e0238191. doi: 10.1371/journal.pone.0238191 32925960 PMC7489558

[8] Akombi BJ, Agho KE, Renzaho AM, Hall JJ, Merom DR. Trends in socioeconomic inequalities in child undernutrition: Evidence from Nigeria Demographic and Health Survey (2003 - 2013). PLoS One. 2019;14(2):e0211883. doi: 10.1371/journal.pone.0211883 30730946 PMC6366715

[9] Odozi JC, Oyelere RU. Evolution of Inequality in Nigeria: A Tale of Falling Inequality, Rising Poverty, and Regional Heterogeneity. Journal of Economics, Race, and Policy. 2023;6(4):297–309. doi: 10.1007/s41996-023-00129-9

[10] Okoli CI, Hajizadeh M, Rahman MM, Khanam R. Geographic and socioeconomic inequalities in the survival of children under-five in Nigeria. Sci Rep. 2022;12(1):8389. doi: 10.1038/s41598-022-12621-7 35590092 PMC9120155

[11] Faust L, Yaya S, Ekholuenetale M. Wealth inequality as a predictor of HIV-related knowledge in Nigeria. BMJ Glob Health. 2017;2(4):e000461. doi: 10.1136/bmjgh-2017-000461 29333285 PMC5759704

[12] Nevo CM, Egenti S. A Disaggregated Analysis of Wealth Status and Educational Attainment in Nigeria Using the Multinomial Logit Approach. Economies. 2019;7(2):39. doi: 10.3390/economies7020039

[13] Berde AS, Yalcin SS. Determinants of early initiation of breastfeeding in Nigeria: a population-based study using the 2013 demograhic and health survey data. BMC Pregnancy Childbirth. 2016;16:32. doi: 10.1186/s12884-016-0818-y 26852324 PMC4744410

[14] Skoufias E, Vinha K. Child stature, maternal education, and early childhood development in Nigeria. PLoS One. 2021;16(12):e0260937. doi: 10.1371/journal.pone.0260937 34941902 PMC8700053

[15] Ohia DrB-F. Girl Child Education in Nigeria as Imperative for Women Empowerment: A Feminist Critique. JWES. 2024;(44):1–11. doi: 10.55529/jwes.44.1.11

[16] Adeyanju O, Tubeuf S, Ensor T. Socio-economic inequalities in access to maternal and child healthcare in Nigeria: changes over time and decomposition analysis. Health Policy Plan. 2017;32(8):1111–8. doi: 10.1093/heapol/czx049 28520949

[17] Udo RK. Geographical Regions of Nigeria. Berkeley, CA: University of California Press. 2023.

[18] Jaiyeola AO, Choga I. Assessment of poverty incidence in Northern Nigeria. Journal of Poverty. 2020;25(2):155–72. doi: 10.1080/10875549.2020.1783424

[19] Ameye H, De Weerdt J. Child health across the rural–urban spectrum. World Development. 2020;130:104950. doi: 10.1016/j.worlddev.2020.104950

[20] Ibitoye JO, Asaolu O, Amao A, Obembe O, Ijaya MA, Obanubi C, et al. A cross-sectional assessment of expanding basic healthcare services to rural and underserved communities through proprietary patent medicine vendors in Northern Nigeria. PLOS Glob Public Health. 2024;4(9):e0003671. doi: 10.1371/journal.pgph.0003671 39302941 PMC11414903

[21] United Nations Children’s Fund (UNICEF). The State of the World’s Children 2024: The Future of Childhood in a Changing World. UNICEF. 2024. https://www.unicef.org/reports/state-of-worlds-children/2024

[22] Link BG, Phelan J. Social conditions as fundamental causes of disease. J Health Soc Behav. 1995;Spec No:80–94. doi: 10.2307/2626958 7560851

[23] Langa N, Bhatta T. The rural-urban divide in Tanzania: Residential context and socioeconomic inequalities in maternal health care utilization. PLoS One. 2020;15(11):e0241746. doi: 10.1371/journal.pone.0241746 33166310 PMC7652341

[24] Clouston S, Kidman R, Palermo T. Social inequalities in vaccination uptake among children aged 0-59 months living in Madagascar: an analysis of Demographic and Health Survey data from 2008 to 2009. Vaccine. 2014;32(28):3533–9. doi: 10.1016/j.vaccine.2014.04.030 24814558

[25] National Population Commission [Nigeria], I C F ICF. Nigeria Demographic and Health Survey 2024. Abuja, Nigeria, and Rockville, Maryland, USA: NPC and ICF. 2024.

[26] Adewusi AO, Nwokocha EE. Maternal Education and Child Mortality in Nigeria. NJSA. 2018;16(1). doi: 10.36108/njsa/8102/61(0170

[27] Clouston SEAP, Yukich J, Anglewicz P. Social Inequalities in Malaria Knowledge, Prevention and Prevalence among Children under 5 Years Old and Women Aged 15–49 in Madagascar. Malaria Journal. 2015;14(1):499. doi: 10.1186/s12936-015-1010-y PMC4676822 26651615

